# Complete mitochondrial genome of the Endangered fish *Anabarilius liui yalongensis* (Teleostei, Cyprinidae, Cultrinae)

**DOI:** 10.1080/23802359.2018.1535850

**Published:** 2018-11-21

**Authors:** Liwen Zeng, Kun Yang, Anxiang Wen, Meng Xie, Yongfang Yao, Huailiang Xu, Guangxiang Zhu, Qin Wang, Yanzhi Jiang, Tao He, Jiayun Wu

**Affiliations:** a College of Life Sciences, Sichuan Agricultural University, Ya'an, PR China;; b Institute of Ecology, China West Normal University, Nanchong, PR China

**Keywords:** *Anabarilius liui yalongensis*, mitochondrial genome, phylogenetic analysis

## Abstract

We describe the complete mitochondrial genome of the Endangered fish *Anabarilius liui yalongensis*. It is a circular molecule of 16,608 bp in size, and all genes show the typical gene arrangement conforming to the vertebrate consensus. The cytochrome c oxidase subunit I (COI) sequence of *A. liui yalongensis* and other 21 species from 14 genera were used for phylogenetic analysis by Bayesian inference and maximum likelihood methods. The topology demonstrated that the *A. liui yalongensis* clustered with *A. grahami* are closely to Hemiculter branch within the subfamily Cultrinae.

*Anabarilius liui*, which belongs to the genus Anabarilius within the subfamily Cultrinae (Teleostei, Cyprinidae), was mainly distributed in Sichuan and Yunnan Provinces of China (Ding 1994). Four subspecies included *A. liui liui, A. liui chenghaiensis*, *A. liui yiliangensis* and *A. liui yalongensis* were detected in *A. liui* (Li and Chen 2003). The *A. liui yalongensis*, which is endemic in the main stream and tributaries of the Yalong River, is special for the body height, stem height, eye diameter and space, lateral line scales, stem tail scale, branched rays of the anal fin and aill raker. The morphically differences between *A. liui yalongensis* and the other three subspecies may be caused by the special bait organisms and the cold environment (Li and Chen 2003). Because of habitat degradation, exotic species invasion and overfishing, the wild resources of the species were sharply decreased in recent years. Now it has been rated as EN (Endangered) in the Chinese vertebrate red list (Jiang et al. 2016).

In this study, we determined the complete mtDNA sequence of *A. liui yalongensis*. The samples were collected from the reservoir area of Jinping hydroelectric station of the Yalong River during October 2016. After sampling, the specimens were stored in 90% ethanol and kept in the natural sciences Museum of Sichuan Agricultural University. The genomic DNA was isolated from fin tissue by the standard phenol–chloroform extraction procedure (Sambrook and Russell 2001). Twenty primer sets were designed to amplify the segments. The complete mitochondrial genome sequence of *A. liui yalongensis* is 16,608 bp in length (GenBank accession no. MG702493), containing 13 protein-coding genes (PCGs), 22 transfer RNA (tRNA) genes, two ribosomal RNA (rRNA) genes, one replication origin (O_L_) and one control region (D-loop). All genes show the typical gene arrangement conforming to the vertebrate consensus (Noack et al. 1996). Among the 13 protein-coding genes, except ND2, COX2, ATP6, and CYTB use an incomplete stop codon ‘T’, the rest are encoded by the typical TAA or TAG stop codons. The single non-coding control region (D-Loop) is 927 bp in length.

The complete mitochondrial genome sequence of the genus Anabarilius is really limited, and the only report was *Anabarilius grahami* (Wang et al. 2017). The cytochrome c oxidase subunit I (COI) sequence of *A. liui yalongensis* and other 21 species from 14 genera were used for phylogenetic analysis by Bayesian inference and maximum likelihood methods in this study. Yue and Luo (1996) divided the subfamily Cultrinae into three groups: Cultrine, Rasborine and Anchidaniorine, and the genera Anabarilius and Sinibrama were both gathered in Rasborinus branch. However, other studies separated the Anabarilius and Sinibrama in Hemiculter and Parabramis branches (Xie 2003; Feng et al. 2009). Consistent with the latter studies, topology of the phylogenetic tree in this study (Figure 1) divided the 22 fishes into three groups (Hemiculter, Culter and Parabramis branches). The genera Anabarilius and Sinibrama were divided into Hemiculter and Parabramis branches. The results of this study confirmed the genetic relationship between the genera Anabarilius and Sinibrama.

**Figure 1.**
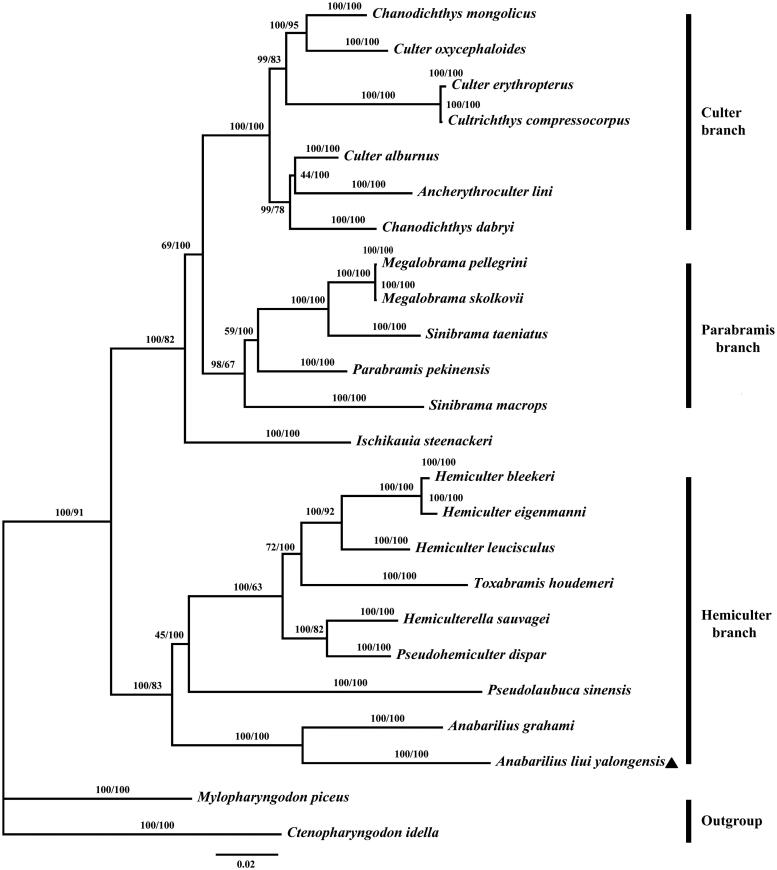
Phylogenetic tree of Cultrinae based on BI and ML method of cytochrome c oxidase subunit I. The bootstrap values for the BI and ML analysis are shown on the nodes (left is BI bootstrap values). Note the Triangle sign represents the species in this study.
